# Supplementary material for: Disparities in pre-health advising across California’s public universities
Source: PLoS One. 2024 Feb 9;19(2):e0296741. doi: 10.1371/journal.pone.0296741 (PMC10857704; doi:10.1371/journal.pone.0296741)
Supplement: S1 Table — (DOCX) [file pone.0296741.s001.docx]

**S1 Table. Additional Campus Data**

| **Campus** | **Graduates/FTE Pre-health Advising** | **Office Structure** |
| --- | --- | --- |
| California State University Campuses – Mean: 24,620 | | |
| CSU #1 | N/A | No pre-health advising |
| CSU #2 | N/A | No pre-health advising |
| CSU #3 | N/A | No pre-health advising |
| CSU #4 | 150,520 | Career Center |
| CSU #5 | 70,517 | Professor |
| CSU#6 | 31,929 | Professor |
| CSU #7 | 28,720 | Professor |
| CSU #8 | 28,557 | Professor |
| CSU #9 | 26,950 | Professor |
| CSU #10 | 26,329 | Professor |
| CSU #11 | 25,000 | Career Center |
| CSU #12 | 18,524 | General Academic Advising |
| CSU #13 | 8,914 | Independent Office |
| CSU #14 | 8,499 | General Academic Advising |
| CSU #15 | 8,185 | Independent Office |
| CSU #16 | 7,300 | Professor |
| CSU #17 | 2,173 | General Academic Advising |
| CSU #18 | 1,059 | General Academic Advising |
| University of California Campuses – Mean: 4,526 | | |
| UC #1 | 10,920 | Career Center |
| UC #2 | 7,147 | Career Center |
| UC #3 | 5,096 | Career Center |
| UC #4 | 4,709 | Independent Office |
| UC #5 | 4,198 | Career Center |
| UC #6 | 2,347 | Independent Office |
| UC #7 | 2,330 | General Academic Advising |
| UC #8 | 2,072 | General Academic Advising |
| UC #9 | 1,912 | Independent Office |
| Private Campuses – Mean: 1,749 | | |
| Private #1 | 5,300 | Professor |
| Private #2 | 1,752 | Independent Office |
| Private #3 | 1,026 | Independent Office |
| Private #4 | 908 | Independent Office |
| Private #5 | 787 | General Academic Advising |
| Private #6 | 722 | Career Center |
