# Supplementary material for: Disparities in pre-health advising across California’s public universities
Source: PLoS One. 2024 Feb 9;19(2):e0296741. doi: 10.1371/journal.pone.0296741 (PMC10857704; doi:10.1371/journal.pone.0296741)
Supplement: Attachment — Submitted filename: Response to Reviewers Pre-health Advising. 12.5.docx [file pone.0296741.s002.docx]

Response to Reviewers: PONE-D-23-24270

“Disparities in Pre-Health Advising Across California’s Public Universities”

Francine Rios-Fetchko, Mariam Carson, MS, Manuel Tapia, MD, MPH, Alicia Fernandez, MD, and Janet Coffman, PhD, MPP

| **Editor/Reviewer Comment** | **Response** |
| --- | --- |
| **Editor** | |
| More details regarding the use of grounded theory are warranted (page 6, lines 145 – 153).  Describe grounded theory and what was done.  Provide the processes involved in conducting this analysis.  For example, Reviewer 1 questioned “whether the authors really do line-by-line coding with no presumptions of what they might find.”  What strategies were used in analyzing the data (e.g., deductive and/or inductive strategies; abductive analysis such as Tavory and Timmermans, 2014). More clarity on the analytic technique and citation to the closest methodological approach would strengthen the article. | Thank you for this comment.  **Lines 146-154 now read:**  Interviews were analyzed using an inductive approach, consistent with modified Grounded Theory approaches. Two research analysts read a random sample of 8 transcripts and generated a preliminary codebook using Jamboard, a tool for visualizing and grouping recurring themes. This codebook was iteratively refined through discussions with the entire research team and applied to the remaining transcripts using Dedoose software to facilitate line-by-line coding. Themes were finalized through iterative discussions among the entire research team. |
| Define the term UIM before using it. It is first mentioned on page 14, line 308.   Indicate what the letters stand for and who is included. | Thank you for this reminder. We have added the following description to address this comment, **lines 327 – 333:**  Many advisors also noted challenges that students from backgrounds historically underrepresented in medicine (UIM) commonly faced, including lack of access to clinical volunteer opportunities, timely early advising, and multiple compounding barriers related to economic and familial pressures. Students considered to be UIM include those who identify as: Black/African American, Hispanic/Latino, Native American (American Indians, Alaska Natives, and Native Hawaiians), Pacific Islander, and mainland Puerto Rican as well as students from economically or educationally disadvantaged backgrounds. |
| Given that data from 3 different types of university systems are compared (i.e., CSU vs UC, vs private schools), the reviewers recommend that for each of the identified themes, examples from each of the university systems be included.  This allows the reader to have a greater understanding of institutional variability or commonality.   For example, for the theme “Competing Priorities for Advising” a reviewer noted that “there was not a discussion of institutional variation. And the quote provided from a CSU campus advisor is a phenomenon that may occur more frequently at public schools with first-generation and low-income students. “ | We now include the following additional examples to highlight comparisons between university systems.  Theme: Insufficient Institutional Investment in Advising  Added l**ines 229 - 232**: One advisor from a private institution described his institution as “a business,” and explained that “ unless you can convince the administration that if you don’t put some money in the pre-health advising, you’re actually going to dimmish the way you look to your customer’s base. I don’t think they’re going to do it.”  Theme: Competing priorities for advising  Added **lines 252 - 254**: Advisors at some UCs also noted institutional pressures to prioritize fulfillment of undergraduate major requirements over other interests, although such comments were less common. No private institutions noted institutional pressure related to increasing graduation rates.  Theme: Advisor turnover and training  Added **lines 304 – 309:** In contrast, one advisor at a private university highlighted their office’s ability to retain staff as a strength of their advising system. They explained:  We have quite a large staff. We have a group where many of our staff have been with the office since its inception 11 years ago. I think we're really able to come together and find creative ways to support the students. I think that the staffing that we have allows us to do that. We also have a decent budget. – private school advisor  **Lines 323 – 325:** On the other hand , schools with independent, career center or general academic advising structures less often cited lack of training as a challenge, with some even pointing toward their expertise in the field as an asset for students.  Theme: UIM Students Face Complex and Disproportionate Challenges  Added **lines 360 – 362:** Advisors from all three university systems noted that UIM students at their campuses face disproportionate challenges, however, discussions of such challenges were more salient at the CSUs due to the high number of UIM students they serve. |
| It is agreed that Table 3 provides an excellent example of potential innovations in pre-health advising.  Reviewer 1 cautions it may be useful to note potential limitations for some of the recommendations.  For example, peer advisors who serve as near-peer advisors sitting in for trained staff may provide inadequate or incorrect information to pre-health advisees. It is useful to note in this table, as was done for the “Committee letter”. | Thank you for this comment. We have added in the following limitations to Table 3:  Peer advisors:  Although peer advisors can be a good resource, they should not take the place of professional advisors and should be supervised closely due to their lack of experience and training in the field.  Connections with local physicians:  Advisors noted that shadowing opportunities have been limited since COVID-19 restrictions were put in place.  Student club partnerships:  Advisors noted that rapid turnover of club leadership poses a challenge to continuity of partnerships. |
| Overall, the topic is very interesting and has the potential to address factors associated with advising and racial/ethnic disparities in the health workforce. You are commended for your attempt to add to the extant literature on the topic of advising pre-health students.  While the study focuses exclusively on pre-health students in California, it has implications for advising for all students attending institutions of higher education. | Thank you very much for this comment, we appreciate your acknowledgement of the importance of this topic and hope this study will have implications for advising nationally. |
| **Reviewer #1** | |
| This study provides a concrete review of pre-health advising disparities in the state of California. CA is a useful case study because the implementation of Prop 209 in 1996 effectively did away from affirmative action for racially marginalized students. In the wake of the Supreme Court decision in Students for Fair Admissions, Inc. v. Harvard College, there are strong reasons to anticipate that pre-health students of color around the country will attend less selective institutions than they might have otherwise. The study attempts to gauge what kind of resources these students will find at less selective institutions. Resources matter for successful movement into health disciplines. Indeed, we see in CA huge differences between the racial background of state residents and representation of Latinx health providers. | Thank you very much for this comment, we appreciate your acknowledgement of the importance of this topic and hope this study will have implications for advising nationally, especially given the recent SCOTUS decision. |
| I am grateful to see clear information and comparisons across sectors in the state and find this article to be quite helpful in documenting disparities. The framing for why the problem is worthy of study is effective. | Thank you for this comment and for letting us know that the framing of the problem was effective. |
| Define UIM before using the term. Indicate what the letters stand for and who is included. | Please see response to editor’s comments. |
| Scholars frequently say they are doing grounded theory when they are not. Did the authors really do line-by-line coding with no presumptions of what they might find? My guess is that this article utilized more movement between deductive and inductive strategies (abductive analysis, Tavory and Timmermans 2014). Please provide a bit more clarity on the analysis technique and a citation to the closest methodological approach. | Please see response to editor’s comments. |
| Within each point, the authors need to give a sense of how common this was at CSU, vs. UC, vs. privates. They often do this well. However, on the “Competing Priorities for Advising” there was not a discussion of institutional variation. And the particular quote provided from a CSU campus advisor is a phenomenon that occurs, I would assume, much more frequently at public schools with first-gen and low-income students. Please contextualize. | Please see response to editor’s comments. |
| I love the table of potential innovations but note caution with peer advisors. I have also see cases where peer advisors sitting in for trained staff provide inadequate or incorrect information and are a poor band-aid for limited pre-health advising. It is useful to note in this table potential limitations of some of these remedies (as is done for the committee letter). | Please see response to editor’s comments. |
| **Journal Requirements** | |
| Please ensure that your manuscript meets PLOS ONE's style requirements, including those for file naming. | Manuscript meets PLOS One style requirements. |
| Note from Emily Chenette, Editor in Chief of PLOS ONE, and Iain Hrynaszkiewicz, Director of Open Research Solutions at PLOS: Did you know that depositing data in a repository is associated with up to a 25% citation advantage ([https://doi.org/10.1371/journal.pone.0230416)?](https://urldefense.com/v3/__https:/doi.org/10.1371/journal.pone.0230416)?__;!!LQC6Cpwp!tCcXyDLYH4kitTF8dcdmvkSFHxB1j_06iL4s1LctDBeZJkBG9C8ZB53_53Uw0yrFmlnri4vlwGK160bEDWRYOtykcGok$) If you’ve not already done so, consider depositing your raw data in a repository to ensure your work is read, appreciated and cited by the largest possible audience. You’ll also earn an Accessible Data icon on your published paper if you deposit your data in any participating repository ([https://plos.org/open-science/open-data/#accessible-data](https://urldefense.com/v3/__https:/plos.org/open-science/open-data/*accessible-data__;Iw!!LQC6Cpwp!tCcXyDLYH4kitTF8dcdmvkSFHxB1j_06iL4s1LctDBeZJkBG9C8ZB53_53Uw0yrFmlnri4vlwGK160bEDWRYOlhSl_rr$)). |  |
| Please state what role the funders took in the study.  If the funders had no role, please state: "The funders had no role in study design, data collection and analysis, decision to publish, or preparation of the manuscript." If this statement is not correct you must amend it as needed. Please include this amended Role of Funder statement in your cover letter; we will change the online submission form on your behalf. | The following was added to the cover letter: The funders had no role in study design, data collection and analysis, decision to publish, or preparation of the manuscript. |
| We note that you have provided funding information that is currently declared in your Funding Statement. However, funding information should not appear in the Acknowledgments section or other areas of your manuscript. We will only publish funding information present in the Funding Statement section of the online submission form.  Please remove any funding-related text from the manuscript and let us know how you would like to update your Funding Statement. | All funding related text was removed from the acknowledgements section and manuscript (formerly line 456). |
| In your Data Availability statement, you have not specified where the minimal data set underlying the results described in your manuscript can be found. PLOS defines a study's minimal data set as the underlying data used to reach the conclusions drawn in the manuscript and any additional data required to replicate the reported study findings in their entirety. All PLOS journals require that the minimal data set be made fully available. For more information about our data policy, please see [http://journals.plos.org/plosone/s/data-availability](https://urldefense.com/v3/__http:/journals.plos.org/plosone/s/data-availability__;!!LQC6Cpwp!tCcXyDLYH4kitTF8dcdmvkSFHxB1j_06iL4s1LctDBeZJkBG9C8ZB53_53Uw0yrFmlnri4vlwGK160bEDWRYOs0qaRLz$).  Upon re-submitting your revised manuscript, please upload your study’s minimal underlying data set as either Supporting Information files or to a stable, public repository and include the relevant URLs, DOIs, or accession numbers within your revised cover letter. For a list of acceptable repositories, please see [http://journals.plos.org/plosone/s/data-availability#loc-recommended-repositories](https://urldefense.com/v3/__http:/journals.plos.org/plosone/s/data-availability*loc-recommended-repositories__;Iw!!LQC6Cpwp!tCcXyDLYH4kitTF8dcdmvkSFHxB1j_06iL4s1LctDBeZJkBG9C8ZB53_53Uw0yrFmlnri4vlwGK160bEDWRYOt0IfmXt$). Any potentially identifying patient information must be fully anonymized.  Important: If there are ethical or legal restrictions to sharing your data publicly, please explain these restrictions in detail. Please see our guidelines for more information on what we consider unacceptable restrictions to publicly sharing data: [http://journals.plos.org/plosone/s/data-availability#loc-unacceptable-data-access-restrictions](https://urldefense.com/v3/__http:/journals.plos.org/plosone/s/data-availability*loc-unacceptable-data-access-restrictions__;Iw!!LQC6Cpwp!tCcXyDLYH4kitTF8dcdmvkSFHxB1j_06iL4s1LctDBeZJkBG9C8ZB53_53Uw0yrFmlnri4vlwGK160bEDWRYOrydcbIZ$). Note that it is not acceptable for the authors to be the sole named individuals responsible for ensuring data access.  We will update your Data Availability statement to reflect the information you provide in your cover letter. | We have added the following data availability statement to the cover letter:  In order to protect participant privacy, transcripts from this study will not be made publicly available. Deidentified quotes from participant interviews have been included in the manuscript, all other data sharing would violate participant consent agreements approved by the University of California, San Francisco Institutional Review Board (Study # IRB # 21-34656). Requests to review additional transcript excerpts should be directed to the UCSF IRB, reachable at (415) 476-1814. |
| We notice that your supplementary table (Appendix A) is included in the manuscript file. Please remove them and upload them with the file type 'Supporting Information'. Please ensure that each Supporting Information file has a legend listed in the manuscript after the references list. | Appendix A has been removed from the manuscript and added as a supplementary file.  A legend reading “ Supporting Information, Table 4. Additional Campus Data” has been added to the manuscript following the references. |
| Please review your reference list to ensure that it is complete and correct. If you have cited papers that have been retracted, please include the rationale for doing so in the manuscript text, or remove these references and replace them with relevant current references. Any changes to the reference list should be mentioned in the rebuttal letter that accompanies your revised manuscript. If you need to cite a retracted article, indicate the article’s retracted status in the References list and also include a citation and full reference for the retraction notice. | The reference list has been reviewed and updated. No retracted references are included.  Reference #23 has been added to address one of the reviewer comments as described in above in this table. |
